# Natalizumab Significantly Improves Cognitive Impairment over Three Years in MS: Pattern of Disability Progression and Preliminary MRI Findings

**DOI:** 10.1371/journal.pone.0131803

**Published:** 2015-07-06

**Authors:** Flavia Mattioli, Chiara Stampatori, Fabio Bellomi, Cristina Scarpazza, Ruggero Capra

**Affiliations:** 1 Neuropsychology Unit, Spedali Civili of Brescia, Brescia, Italy; 2 Multiple Sclerosis Center of the Spedali Civili of Brescia, Montichiari, Italy; Friedrich-Alexander University Erlangen, GERMANY

## Abstract

Previous studies reported that Multiple Sclerosis (MS) patients treated with natalizumab for one or two years exhibit a significant reduction in relapse rate and in cognitive impairment, but the long term effects on cognitive performance are unknown. This study aimed to evaluate the effects of natalizumab on cognitive impairment in a cohort of 24 consecutive patients with relapsing remitting MS treated for 3 years. The neuropsychological tests, as well as relapse number and EDSS, were assessed at baseline and yearly for three years. The impact on cortical atrophy was also considered in a subgroup of them, and are thus to be considered as preliminary. Results showed a significant reduction in the number of impaired neuropsychological tests after three years, a significant decrease in annualized relapse rate at each time points compared to baseline and a stable EDSS. In the neuropsychological assessment, a significant improvement in memory, attention and executive function test scores was detected. Preliminary MRI data show that, while GM volume did not change at 3 years, a significantly greater parahippocampal and prefrontal gray matter density was noticed, the former correlating with neuropsychological improvement in a memory test. This study showed that therapy with Natalizumab is helpful in improving cognitive performance, and is likely to have a protective role on grey matter, over a three years follow-up.

## Introduction

In recent years, several new drugs have been approved for the treatment of relapsing remitting multiple sclerosis (RRMS) [1]. Within the new drugs, the natalizumab (Tysabri, Biogen, Cambridge, MA) has been shown to have a large effect in reducing disease activity. In particular, studies from our and other groups reported that patients under treatment with natalizumab for a period of time between 6 months and 2 years exhibit a significant reduction in the decay and, interestingly, even an improvement in cognitive functions, particularly in information processing, executive functions and memory domains [2–9]. Notably, recent studies showed that, after one year of administration, treatment with natalizumab was able to inhibit the accumulation of cortical lesions and the progression of cortical atrophy with a significantly higher effect compared to other immunomodulatory therapies [9,10]. Natalizumab treatment considerably targets grey matter and these findings may likely represent the anatomical background for its impact on cognitive functions, which are strongly associated with cortical pathology [11–15].

Several studies, for instance the TOP (Tysabri Observational Program [16]), confirmed the natalizumab driven overall safety profile, low relapse rate and stabilized disability levels in treated patients over 5 years. However, no long term effects of natalizumab on cognitive functions have to date been described beyond the second year of treatment. Indeed, the TOP study used the Expanded Disability Status Scale (EDSS) total score as outcome index that, although it is comprehensive of a cognitive score too, it has little sensitivity to cognitive functioning.

Since in the previous literature studies no neuropsychological data were provided beyond the second year of therapy, we aimed in investigating the evolution of cognitive impairment over time in patients treated with natalizumab. To this end, we performed an observational study on neuropsychological functions of a group of relapsing remitting MS treated with natalizumab for three years and, in a subgroup of them, we analyzed their correlates with brain atrophy measured with MRI.

## Materials and Methods

### Patients selection

24 RR-MS consecutive patients, treated with natalizumab according to Italian Medicines Agency (AIFA) reimbursement criteria [17] starting on April 2007, were observed for three years at the MS Center of the Spedali Civili of Brescia. Their baseline socio-demographic information are reported in Table 1. They were all naive to natalizumab therapy and did not have any therapy interruption during the three years follow up. Two of them were previously treated with copolymer, 14 of them with interferon B and 4 of them with both copolymer and interferon. Each patient signed an informed written consent before treatment initiation; they also gave consent for their clinical records to be used in the current study. The study was retrospective and observational, authorized by the Ethical Committee of the Spedali Civili of Brescia and in conformity with the Helsinki Declaration.

**Table 1:** Baseline socio-demographic information.

	*Age*	*Education*	*Gender*	*Disease Duration*
*Mean*	36.8	12.3	13 ♀	12.15
*SD*	9.04	3.1	11 ♂	6.0

The table reported mean and standard deviation. Age, education and disease duration are expressed in years.

♀ = female

♂ = male.

Patients were evaluated according to the good clinical practice procedure; for the present study, four evaluations were considered: baseline evaluation (i.e., before natalizumab treatment), after one year, 2 years and 3 years of treatment. A clinical examination (EDSS and relapse rate) and the neuropsychological assessment were performed for each patient from different investigators at different time points, blinded of previous patient's clinical condition.

### Neuropsychological evaluation

A wide panel of neuropsychological tests investigating verbal and non-verbal memory, sustained attention, information processing, conceptual reasoning, verbal fluency, and executive functions were used: see also [4,5] for tests references). All participants underwent a neuropsychological examination to test the general cognitive functioning through several widely used neuropsychological tests. The neuropsychological tests included: the Raven's Colored Progressive Matrices PM47 (CPM) [18,19], to test intellectual skills that do not depend on verbal skills; the Digit Span forward [20], to assess the short-term verbal memory; the Short Tale [21], to evaluate the long-term verbal memory; the Corsi block tapping Test [20,22,23], to test the short-term spatial memory; the Rey-Osterrieth Complex Figure Test (ROCFT) [24–26], to investigate constructional praxis, visuographic memory, and some aspects of planning and executive functions, with immediate and delayed recall; the controlled oral word association test with phonemic (COWA/P) and semantic cues (COWA/S) [27–29], to assess the lexical stock and the ability to access to the lexicon; the Paced Auditory Serial Addition Test (PASAT, both 2” and 3” interval) [30], to evaluate working memory, information processing, and sustained attention; the Wisconsin Card Sorting Test (WCST) [31], to test the abstract reasoning, decision-making, planning, conceptual shift, learning new rules, and the ability to change cognitive strategies to environmental conditions changing. WCST total errors (te), perseverative responses (pr) and perseverative errors (pe) were assessed.

Alternative forms of both verbal and non-verbal tests, when available, were employed in order to avoid a learning effect. Tests were performed in a quiet environment according to published procedures. A score < -2.0 Z-score below the corresponding control mean was considered as impaired (failed test).

### MRI Acquisition and analysis

Baseline and three years later MRI exams, performed in the same place with the identical procedure, were compared in a subgroup of 9 patients.

#### MRI acquisition

(MR magnet Siemens Avanto 1.5 T): Turbo Spin Echo and FLAIR T2W, Spin Echo T1 W, contiguous 3 mm axial slices and FLAIR 3 mm sagittal slices images were obtained.

#### Preprocessing

Each MRI was checked for scanner artifacts, reoriented along the anterior-posterior commissure (AC-PC) line and the AC was set the AC as the origin of the spatial coordinates. Images were segmented into gray matter (GM) and white matter (WM) using the new segmentation procedure implemented in SPM8 (http://www.fil.ion.ucl.ac.uk/spm), running under Matlab 7.1 (Math Works, Natick, MA, USA). A fast diffeomorphic image registration algorithm (DARTEL;[16]) was used to warp the GM partitions into a new study-specific reference space representing an average of all the subjects included in the analysis [32,33]; the warped GM partitions were affine transformed into the MNI space and smoothed with a 8-mm full-width at half-maximum (FWHM) Gaussian kernel. An additional ‘modulation’ step was used in order to ensure that the total amount of gray matter in each voxel was conserved after the registration [34–36]. After this preprocessing, we obtained smoothed, modulated, normalized data that were used for the statistical analysis.

### Statistical analysis

Clinical data are expressed by descriptive analysis as mean annualized relapse rate (ARR), mean EDSS and percentage of patients free from clinical disease activity [(i.e., no relapses and no disability progression as measured by EDSS [37] at given time points (baseline and after 1, 2 and 3 years of treatment). Data from 1, 2 or 3 years of observation are compared to corresponding values at baseline and any previous time point by means of repeated measures Analysis of Variance (ANOVA) using Time (4 levels: baseline, year 1, year 2 and year 3) as a within subjects variables. Newman-keuls post hoc test was used when necessary.

Data are also analyzed by stratifying patients according to medical history parameters (baseline EDSS score) in order to evaluate the presence of baseline factors predicting the response to natalizumab [38]. Patient with EDSS score < 3 (lower neurological impairment) were 11, while patients with EDSS score > 3 (higher neurological impairment) were 13.

Results of cognitive assessments are expressed by descriptive analysis as mean scores for single tests at given time points (baseline and at 1, 2, and 3 years). Results of tests at 1, 2, 3 years follow ups are compared to corresponding values at baseline and any previous time point. The number of pathological tests per patient is considered as a measure of general cognitive impairment. Data (row scores) were analyzed by means of repeated measures ANOVA using Time (4 levels: baseline, year 1, year 2 and year 3) as a within subjects variables. Newman-keuls post hoc test was used when necessary.

#### MRI Statistical analysis

Both the GM volume analysis and the VBM analysis has been performed. Regarding the GM volume analysis, GM segmented images were used to calculate GM volume for each subject both in baseline and at follow-up examination. Data were further analyzed using a two paired t test. Moreover, segmented, normalized, modulated and smoothed images were used to carry out a voxel based morphometry analysis. Each single subject's baseline and 3 year scans were compared and 9 pairs of scans were studied. EDSS was entered into the design matrix as a covariate of no interest, to minimize any impact of this variable on the findings. To exclude voxels outside the brain, we used an implicit mask to remove all the voxels whose intensity fell below the 20% of the mean image intensity. To identify regionally specific changes that were not confounded by global differences in gray matter volume, the proportional scaling option has been adopted. A paired-sample t-test was performed to identify significant differences between 3 years follow up and baseline examinations. Statistical inferences were made at voxel-level using two different thresholds: p<0.05 with family-wise error (FWE) correction for multiple comparisons across the whole brain and p<0.001 uncorrected across the whole brain. In addition, an extent threshold of 50 voxels was applied, thus reporting only clusters which comprised of 50 or more voxels.

## Results

Clinical characteristics of 24 patients at baseline, at 1, 2 and 3 years follow ups are reported in Table 2, while their neuropsychological results are reported in Table 3.

**Table 2:** Clinical characteristics of patients at different follow ups.

Years of follow up	0	1	2	3
ARR	2.41	0.27	0.23	0.27
EDSS	4.52	4.61	4.52	4.52
% free from disease activity	0	73.2	71.7	81.8

ARR: annualized relapse rate; EDSS: expanded disability status scale

**Table 3:** Neuropsychological tests scores at different follow ups.

	Baseline	1-year follow up	2-years follow up	3-years follow up	p
**N° failed tests**	2.00 ± 2.17	1.06 ± 1.59	0.67 ± 1.03	0.61 ± 1.46	**0.0002**
**PASAT 2”**	26.81 ± 15.88	31.06 ± 16.10	35.63 ± 15.68	35.44 14.84	**0.0006**
**PASAT 3”**	41.06 ± 18.60	41.06 ± 16.40	47.25 ± 10.43	44.13 ± 15.76	**0.0309**
**WCST, TE**	26.34 ± 18.98	20.72 ± 16.79	19.82 ± 8.80	18.15 ± 16.80	**0.0270**
**WCST, PR**	24.78 ± 15.98	13.76 ± 12.02	11.14 ± 5.85	12.05± 15.49	**0.0001**
**WCST, PE**	19.96 ± 11.87	13.86 ± 10.39	12.51 ± 8.94	11.73 ± 11.74	**0.0014**
**COWA P**	33.94 ± 12.16	37.61 ± 13.38	38.67 ± 12.18	37.06 ± 12.32	0.0547
**COWA S**	42.65 ± 10.71	44.12 ± 10.26	46.76 ± 11.01	46.24 ± 12.01	0.0973
**Short tale (z)**	-0.75 ± 1.39	-0.33 ± 0.97	0.42 ± 1.25	-0.44 ± 1.22	**0.0120**
**Digit Span**	6.13 ± 0.89	6.31 ± 1.45	6.44 ± 1.36	6.38 ± 1.02	0.5756
**Corsi**	5.00 ± 0.97	5.00 ± 0.89	5.25 ± 1.18	5.19 ± 0.98	0.5007
**CPM Raven**	32.18 ± 3.30	32.00 ± 3.72	32.94± 2.79	32.06 ± 3.07	0.2039
**Rey Copy**	32.47 ± 7.19	33.50 ± 3.51	32.85 ± 3.54	32.82 ± 3.76	0.8481
**Rey Recall**	16.47 ± 9.10	19.06 ± 8.88	19.09 ± 10.05	19.68 ± 9.43	**0.0360**

See text for tests abbreviation. Repeated measures ANOVA was used to compare tests mean scores at different follow ups. The p value related to the main effect of the within variable Time is reported in this Table.

### Results on clinical disease activity

A significant ARR decrease was observed (repeated measures ANOVA p = .000) respectively at 1 year follow up (0.27 vs 2.41; p = .000), 2 (0.23; p = .00) and 3 years (0.27; p = .00) compared to baseline, without significant differences between second and third follow ups (p = n.s). EDSS did not change over time (repeated measures ANOVA p = n.s). Patients who, at baseline, had EDSS<3 compared to patients with EDSS>3 did not significantly differ at baseline in ARR (2.50 vs 2.36; p = n.s.), as well as at different follow ups (1 year 0.25 vs 0.29; 2 years 0.13 vs 0.29 and 3 years 0.25 vs 0.29. p = n.s.). Patients free from disease activity were 0% at baseline and 81.82% after three years. Patients who, at baseline, had EDSS<3 compared to patients with EDSS>3 did not significantly differ at baseline in ARR (2.50 vs 2.36; p = n.s.), as well as at different follow ups (1 year 0.25 vs 0.29; 2 years 0.13 vs 0.29 and 3 years 0.25 vs 0.29. p = n.s.).

Both patients starting Natalizumab with EDSS<3 and >3 did not significantly change in EDSS over time (mean EDSS at 1 year 1.81, at 2 years 1.63 and at 3 years 1.50 in <3 EDSS; mean EDSS at 1 year 6.21, at 2 years 6.18 and at 3 years 6.25 in >3 EDSS).

### Neuropsychological evaluation

**Fig 1:**
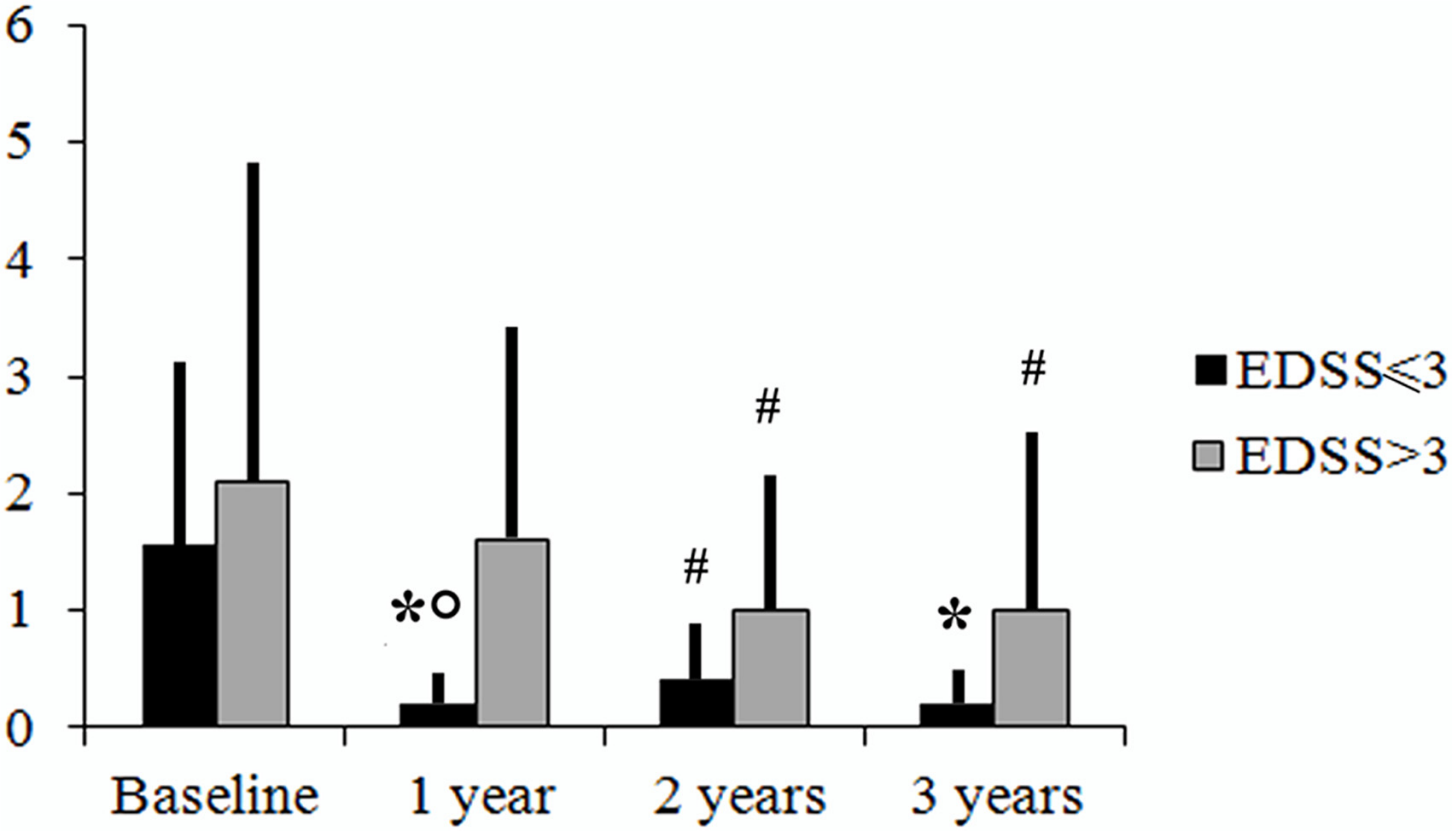
Mean number of impaired tests in patients starting natalizumab with EDSS>3 or<3. Columns represent mean (± SD) number of failed cognitive tests in patients starting natalizumab with EDSS ≤3 (black) or >3 (grey). *, *p*<0.005 *vs*. baseline; #, *p*<0.01 *vs*. baseline; °, *p*<0.05 *vs*. EDSS >3.

Cognitive functions resulted to be globally improved after three years therapy with natalizumab (Table 3). The number of pathological tests resulted significantly lower at 1 year compared to baseline (1.06 vs 2; p = .005), at 2 years compared to baseline (0.67 vs 2; p = .0002) and 3 years compared to baseline (0.61 vs 2, p = .0001), without significant differences in comparisons between the year intervals (p = n.s.). Patients with baseline EDSS <3 compared to patients with baseline EDSS>3 resulted to have a significantly lower number of impaired neuropsychological tests at 1 year (mean number of pathological tests 0.14 vs 1.64; p = .04). No differences were found between groups at subsequent follow ups, although both groups resulted to reduce the number of impaired tests. (Fig 1).

The tests which resulted to be significantly improved over time (repeated measures ANOVA) were PASAT 2", PASAT 3", WCST te, WCSTpe, WCSTpr, Short tale and Rey figure recall (p < .05).

In particular, 1 year vs baseline comparison resulted significant (with higher scores in 1 year follow up) in PASAT 2" (p = .02), WCST te (p = .03), WCST pr (p = .000), WCST pe (p = .006) and Rey figure recall (p = .002). 2 years scores compared to baseline were significantly higher in PASAT 2" (p = .000), PASAT 3" (p = .01), WCST te (p = .01), WCSTpr (p = .000), WCSTpe (p = .001), Short tale (p = .001) and Rey figure recall (p = .002). Also 3 years scores were significantly higher than baseline in PASAT 2" (p = .000), WCST te (p = .002), WCSTpr (p = .000), WCSTpe (p = .000), Rey figure recall (p = .007). On the other hand, 1 year scores compared to 2 years were significantly higher only in PASAT 2" (p = .019), PASAT 3" (p = .01) and Short tale (p = .036); 2 years vs 3 years comparison was significant only in Short tale (p = .07). This pattern shows that the majority of the improvement in most cognitive tests was obtained in the first year of therapy.

Between groups (EDSS<3 or >3 at baseline), comparison at different time points was not different in all the tests, showing that both groups similarly improved mainly in the first year and subsequently stabilized their performances.

### MRI results

#### GM volume results

GM volume at baseline was 904.23±60.85 mm3. GM volume after 3 years was 940.71±42.10 mm3 (p = ns, two paired t test). Data on single subjects examinations reveal that in 7 up to 9 subjects an increase in GM at follow up compared to baseline is found, with a decrease in two out of 9.

#### VBM results

**Fig 2:**
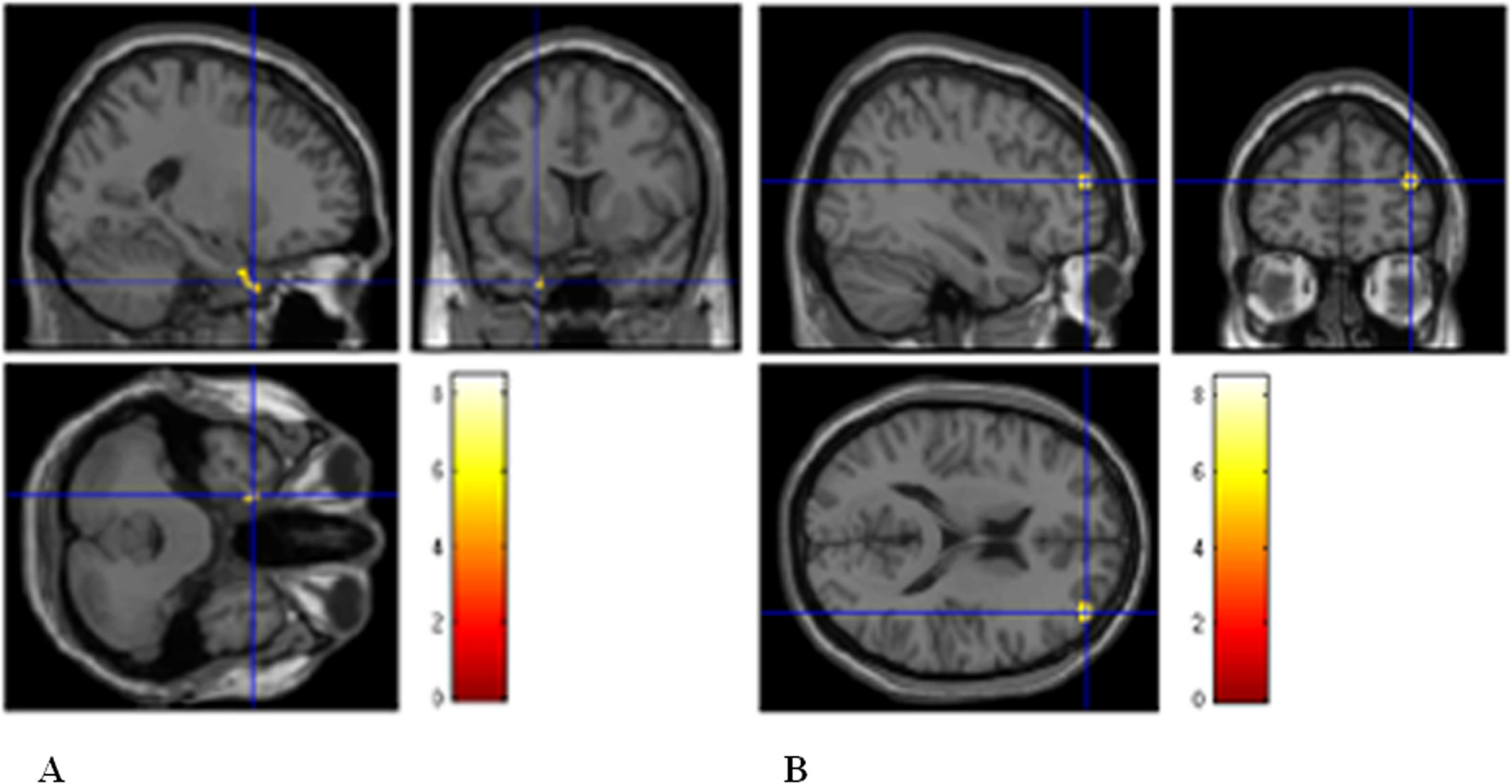
VBM results (follow-up > baseline). The Fig 2 represents: A) the significant increase in parahippocampus GM density, B) the increase GM density in dorsolateral prefrontal cortex as revealed by the longitudinal VBM analysis.

After 3 years, an increase in mean cortical volume compared to baseline in the dorsolateral prefrontal cortex (39 51 15, 143 voxels) and in the parahippocampus (x = -20, y = 5, z = -32,76 voxels) was found (p<0.001 uncorrected). A decreased cortical volume in a small 59 voxel area of the precuneus (x = 0, y = -54, z = 33,59 voxels) was otherwise found. Results are shown in Fig 2.

## Discussion

This study shows that natalizumab is effective in reducing cognitive impairment in MS patients treated for three years: both the number of pathological tests and the single test performances in attention (PASAT), executive functions (WCST) and memory functions (Short tale and Rey figure recall) significantly improve. The improvement results to be substantial in most tests after the first year of therapy. Remarkably, the patients who manifested lower disability at the baseline evaluation (EDSS<3) show a significantly greater improvement in general cognition (number of pathological tests) compared to those ones with higher disability at baseline (EDSS >3), suggesting that the protective effect of this therapy would be greater on mildly disabled cases, who get the greater benefit in terms of cognitive disability progression. Furthermore, compared to EDSS, which is an evaluation tool mostly addressing impaired motor skills, cognitive status and particularly the number of impaired tests, deserves a potential more sensitive prognostic value on the effects of this therapy. Interestingly, the neuropsychological scores at three years follow up resulted to be significantly higher compared to baseline in tests measuring working memory, information processing, sustained attention, flexibility and executive functions. In other words, natalizumab seems to manifest its benefits on neuropsychological performance through the improvement of those functions that are mainly and commonly impaired in MS [2–9]. Several studies addressed the protective effect on cognition of natalizumab over one and two year follow ups [4–8,39], although this is the first one who showed the persistence of these effects also at three years. The effect on cognitive improvement is evident soon at one year and the evolution of clinical disability as well as of cognitive impairment, appears to have a subsequent stabilization. Although the limitation of these data due to the absence of a control group, our data support a significant role of natalizumab in preserving the cognitive functions in MS, which are reported to be impaired in a proportion of one third of MS patients population [40].

The anatomical correlates of the clinical and cognitive improvement of our sample may be the substantial sparing of GM over time, as observed in the sample of patients studied with MRI, whose GM volume did not change after a three year assessment. Interestingly, a significant increase in cortical density was detected in prefrontal and parahippocampal regions. Although these data are to be considered preliminary and need to be replicated on larger population, they suggest that natalizumab have a neuroprotective effect on brain cortical regions from inflammatory and degenerative damage, which has been reported to occur very early in the course of MS [14]. It is likely that the strong anti-inflammatory effect of Natalizumab results in a secondary neuroprotection and allows MS patients to compensate cognitive deficits. In other words, as previous works suggested [41,42,43], brain atrophy in MS might be related to the inflammatory activity (also called pseudoatrophy), and natalizumab shows a secondary neuroprotective role that is probably produced through reducing the inflammatory process in the brain parenchyma. This might results in reduced atrophy and in turn, improved cognitive functioning. A central issue of MS immunomodulatory therapies is their effect in restraining cortical damage from the beginning of disease. Our data support the valuable impact of natalizumab on both cognitive deterioration and cortical grey matter density lasting in a long term period in patients who were already cognitively impaired at the natalizumab beginning and had their impairment reversed shortly in one year. Although a limitation of this study is the lack, due to ethical reasons, of a comparator group of patients with different therapies and the limited sample of MRI studies performed, such an impressive improvement in cognitive performances supports the hypothesis of the indirect effect of the drug on cortical damage.
